# The Evaluation of Personal Protective Equipment Usage Habit of Mining Employees Using Structural Equation Modeling

**DOI:** 10.1016/j.shaw.2022.03.004

**Published:** 2022-03-15

**Authors:** Nilufer Kursunoglu, Seyhan Onder, Mustafa Onder

**Affiliations:** 1 Batman University, Department of Petroleum and Natural Gas Engineering, Batman, Turkey; 2 Eskisehir Osmangazi University, Mining Engineering Department, 26040 Eskisehir, Turkey

**Keywords:** Mining employees, Occupational accidents, Personal protective equipment, Structural equation modeling

## Abstract

**Background:**

In occupational studies, it is a known situation that technical and organizational attempts are used to prevent occupational accidents. Especially in the mining sector, if these attempts cannot prevent occupational accidents, personal protective equipment (PPE) becomes a necessity. Thus, in this study, the main objective is to examine the effects of the variables on the use of PPE and identify important factors.

**Methods:**

A questionnaire was implemented and structural equation modeling was conducted to ascertain the significant factors affecting the PPE use of mining employees. The model includes the factors that ergonomics, the efficiency of PPE and employee training, and PPE usage habit.

**Results:**

The results indicate that ergonomics and employee training have no significant effect (p > 0.05) on the use of PPE. The efficiency of PPE has a statistically meaningful effect (p < 0.05) on the use of PPE. Various variables have been evaluated in previous studies. However, none of them examined the variables simultaneously.

**Conclusion:**

The developed model in the study enables to better focus on ergonomics and employee training in the PPE usage. The effectiveness of a PPE makes its use unavoidable. Emphasizing PPE effectiveness in OHS training and even showing them in practice will increase employees’ PPE usage. The fact that a PPE with high effectiveness is also ergonomic means that it will be used at high rates by the employee.

## 1 Introduction

Mining is a hazardous activity and a miner is considered to be a constant conflict with unpredictable forces of nature as this profession involves working at one of the most hazardous environments in the world. Consequently, mining production lasts to be related to a high level of injuries, accidents, and illnesses [1]. Occupational health and safety is a significant social problem as occupational injuries may cause substantial human suffering and create a significant financial burden to the society [2]. Given the magnitude of the problem, it is important to understand the occupational health and safety approach which mainly focuses on issues related to the behavior of employees [3].

A principal critical phase to develop a detailed safety and health program is to determine physical and health hazards in the workplace. This process is identified as a hazard assessment [4]. Personal protective equipment (PPE) represents clothing or an apparatus worn by employees to keep them from physical impact, fire, or chemicals and shows the latest way of defense [5]. Although the removal of hazard is the first precedence, it has been guessed that human fault is a contributing feature in 84% to 94% of industrial damage cases and one of the most widespread defects is the neglect to wear suitable PPE. Huge declines in occupational injuries and accidents may be attained by rising the occurrence of comparatively modest discretionary safety manners, such as PPE use [2].

Sapbamrer and Thammachai [6] stated that legal obligations are necessary to provide and encourage the use of PPE, and they also emphasized the importance of training that includes the benefits of using PPE. When engineering, work practice and administrative controls are not feasible or do not provide sufficient protection, employers must provide PPE to their employees and ensure its use [4]. Tetzlaff et al. [7] mentioned the use of PPE by employees as an important example of adhering to the safety management system and compliance. López-Toro et al. [8] emphasized that manufacturers in the agriculture and forestry sector should make the use of PPE mandatory both for the safe use of the devices and to reduce injuries that may occur during use. The positive feedback from managers and supervisors plays an important role in promoting the use of PPE [9].

In the mining sector, it has been stated that factors related to the design or maintenance of PPE or safety equipment also contribute to the occurrence of accidents [10]. Serious injuries in underground mining operations can be minimized with the use of appropriate PPE [11]. Zhang et al. [12] stated that the importance of personal protective equipment is overlooked in accidents that occur in coal mining enterprises, as a lack of safety culture. Ismail et al. [13], in their study, examined the ignoring of the use of PPE in the main causes of mining accidents in the category of unsafe behaviors.

According to OSHA, the proper use of PPE can prevent 37.6% of occupational injuries and diseases. 12%–14% of occupational injuries resulting in total disability are caused by employees not wearing appropriate PPE [14]. The use of PPE may lead to discomfort to employees due to sweat, visibility, breathe, or movement problems. These effects need to be fully assessed to predict PPE use. Multiple constituents cause the usage of multivariate models. Structural Equation Modeling which is a multivariate statistical technique can reveal the direct or indirect relations among exploratory factors and a dependent factor [15,16]. Discovering the relations in a single model is important and SEM is proficient to explain this kind of problem [17]. SEM has been substantially used in numerous areas including mining, marketing, ecology, psychology, banking, and safety behavior [[18], [19], [20], [21], [22]].

Several methods such as decision-making process, correlation analysis, regression analysis, principal component analysis, experimental measurement, univariate and bivariate analyses were conducted to evaluate the PPE usage [2,3,9,[23], [24], [25], [26]]. From related literature research [3,9,14,24,27,28], four variables were determined to construct the model. In this study, the main purpose is to examine the effects of these variables on the use of PPE and to identify which of the factors are important.

## 2 Materials and Methods

### 2.1 Study design

**Fig. 1:**
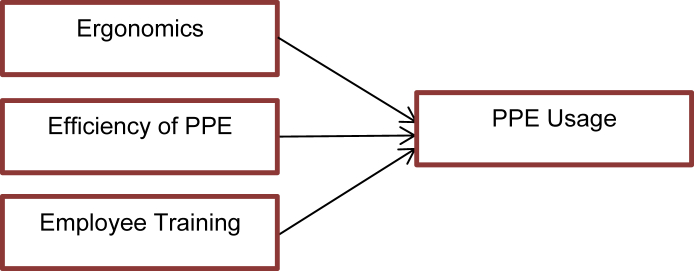
Research framework.

The research model includes four constructs namely ergonomics, employee training, the efficiency of PPE, and the PPE usage habit. The research framework is presented in Fig. 1. The four model constructs were explained below:

1. Ergonomics: PPE must be designed and manufactured to comply with the predictable situations of the use so the intended user can implement the hazard related activity.
2. The efficiency of PPE: PPE should be designed and manufactured in such a manner that it will be easy to stop at the correct position on the user, taking into account the movements to be made and the shape of the body during work, and remain in place for the intended use.
3. Employee training: Training helps employees in obtaining the essential knowledge, skills, and attitude to be capable of using the required PPE [29].
4. PPE usage habit: While examining the various accidents as well as their claims and causes, it is observed that most of these accidents are because employees did not use the required PPE.

### 2.2 Data collection

A questionnaire was developed in the study, and the data were examined. The reliability and validity assessments were conducted for this objective. There are several statistical methods for determining the survey's reliability. This is conducted by evaluating the reliability of the answers to all of the questions on a survey as a whole, as well as the replies to each individual question. Internal consistency is the most commonly used means of determining reliability. The method examines the correlation or link between the responses to items on a survey. The Cronbach's α test was used to determine the measurement items' reliability. Construct validity is a statistical technique for determining if test measures what it claims to measure. Barlett's test of sphericity (BTOS) and Kaiser–Meyer–Oklin (KMO) values were also used to assess the construct validity [30]. The questionnaire was applied to the company’s employees operating in the mining sector in Turkey. Although there were 115 initial participants, 15 questionnaires were excluded due to missing values, and 100 participants' data were used for the final analysis. Tabachnick and Fidell [30] suggested that when the variable number is not too large, sample size between 100 and 200 is sufficient. The participants were asked to answer according to a 5-point Likert scale ranging from “strongly disagree” (1) to “strongly agree” (5). Table 1 represents the demographic characteristics of employees.

**Table 1:** Demographic data of participants

Category	Item	Frequency
Marital status	Single	8
	Married	92
Age	20–25	2
	26–31	5
	32–37	11
	38–43	25
	44–49	28
	50+	29
Level of education	Elementary school	64
	Intermediate school	18
	High school	16
	Graduate	1
	Post graduate	1
Work experience (year)	1–5	32
	6–10	37
	11–15	20
	16–20	2
	20+	9
Sum		100

A pre-test was conducted in the company to (1) aid respondents in their understanding of each item, improve its contents, and texts, (2) minimize ambiguity, and (3) avoid the use of redundant and unneeded measuring indicators. Pre-tests of the initial 20-item questionnaire were carried out with 50 employees. Questions of the survey were presented as detailed in Table 2.

**Table 2:** Questions of the survey

Construct	Indicator	Item
Ergonomics	ERG1	Dust masks make it difficult to breathe during work
	ERG2	Eye spectacles hinder viewing due to lens fogging
	ERG3	Body protectors restricts movements in areas that require active work
	ERG4	PPE are easy to access
	ERG5	Dust masks and eye spectacles fit on face completely
	ERG6	Hearing protectors hinder speaking and hearing
Efficiency of PPE	EP1	Eye protectors protect eyes from various harmful substances and impacts
	EP2	Working clothes are suitable for seasonal conditions
	EP3	Dust masks provide protection completely against fine dusts
	EP4	Hearing protectors reduce the sound pressure level of the noise adequately
	EP5	Body protectors are adequately durable against chemical splash and tearing
	EP6	PPE are integrated with each other
Employee training	ET1	I have information about the risks in work environment
	ET2	I have sufficient training in PPE usage
	ET3	I change PPE periodically
	ET4	I carefully protect and keep all my PPE for my next work
	ET5	I have information about occupational safety and health
PPE usage habit	PUH1	I wear body protectors regularly
	PUH2	I use dust mask and eye protector in all working conditions
	PUH3	I use hearing protection in noisy environment

### 2.3 Data analysis

Exploratory factor analysis (EFA) was implemented on survey data to determine the latent variables or the causal variables under which numerous features were classified. EFA comprises of factor rotation, extraction, and factor loadings. Factor extraction includes the identification of the causal variables that sufficiently describes the observed association between the attributes. There are multiple techniques related to factor extraction in the literature. In the present article, the principal component extraction method was chosen since we aim to reveal the most suitable number of factors that clarify the largest quantity of difference between the studied attributes. The rotation method used in the present article is varimax.

Suitability of the data for EFA was implemented using ’BTOS and KMO measure. The result of the KMO value larger than 0.5 is accepted appropriate for EFA. BTOS having value of *p* < 0.01 is accepted appropriate for EFA [31].

In the literature, there are a variety of views on factor load limit values. The higher the factor weight, the greater the question's ability to describe the relevant factor, and hence the factor's reliability. To say that an item accurately measures a construct or factor, the factor load value must be 0.30 or above. In general, factor loading values of 0.60 and higher, regardless of sign, are regarded "high," whereas loading values of 0.30–0.59 are considered "medium." For different sample sizes, the researcher can use the idea of statistical power to establish factor loadings that are considered significant. Factor loadings of 0.55 and above are significant in a sample of 100 respondents [32]. When an object has a low factor load, it means it is not closely associated with that factor. The following are the results of evaluations based on factor loading values [33]:

• It is considered "good" if the factor load is 0.55 (explaining 30% of the variance).
• It is considered "mediocre" if the factor load is 0.45 (explaining 20% of the variance).
• It is considered "poor" if the factor load is 0.32 (explaining 10% of the variance).

The factor loading of each variable should be at least 0.57. This is the minimum rate declared in the literature and signifies the good association between attributes and latent factors [23]. Factor loadings for this study were assessed to be 0.57 and above, in accordance with the above findings.

Composite reliability (CR) and average variance extracted (AVE) were calculated to assess the validity of the scale. Nunally and Bernstein [34] suggested a value of 0.70 or higher for CR and according to Segars [35], it is accepted sufficient if AVE is 0.50 or higher. The dependability of the constructs was tested using Cronbach Alpha coefficient. Cronbach Alpha evaluates the internal consistency of the factors in data. Cronbach Alpha value of 0.70 or higher is considered acceptable [36]. EFA, KMO, Barlett’s test of sphericity and reliability analysis were performed in the statistical software SPSS 24. Table 3 illustrates the results of the EFA.

**Table 3:** Results of exploratory factor analysis

Construct/Indicator	Items	Factor loading	Cronbach alpha	CR	AVE
Ergonomics			0.929	0.885	0.562
ERG1	I1	0.768			
ERG2	I2	0.816			
ERG3	I3	0.718			
ERG4	I4	0.694			
ERG5	I5	0.701			
ERG6	I6	0.792			
Efficiency of PPE			0.889	0.847	0.526
EP1	I7	0.704			
EP2	I8	0.673			
EP3	I9	0.732			
EP4	I10	0.730			
EP5	I11	0.785			
EP6		∗			
Employee training			0.820	0.807	0.514
ET1	I12	0.783			
ET2	I13	0.616			
ET3	I14	0.754			
ET4	I15	0.705			
ET5		∗			
PPE usage habit			0.883	0.794	0.563
PUH1	I16	0.747			
PUH2	I17	0.761			
PUH3	I18	0.742			

∗ These variables were deleted. The factor loadings of the indicators: EP6: 0.397, ET5: 0.489.

The result of KMO is obtained to be 0.920 and the outcome of BTOS indicates the value of *p* < 0, which means the survey data are suitable for factor analysis. All factor loadings in Table 3 are larger than 0.57, representing an acceptable significant level of internal validity. One question from the efficiency of PPE and one question from employee training was deleted since factor loadings of these variables are below 0.57. The factor loadings of the scale are at an acceptable significant level. Cronbach Alpha values are higher than 0.70 of all constructs, which indicate good reliability of data. CR and AVE values of the constructs are higher than minimum levels of 0.70 and 0.50, respectively.

### 2.4 Confirmatory factor analysis

Confirmatory factor analysis (CFA) is a multivariate statistical methodology utilized to reveal comprehensively a model analyzed by EFA. CFA was used to test the measurement model for each construct and evaluate the relationships between latent variables and their indicators. In this study, CFA was conducted using AMOS 24 software. The analysis was constructed using the maximum likelihood approach. CFA verifies the factor structure using numerous statistical measures that check the goodness of the model fit. Several goodness-of-fit indices suggested by numerous investigators were utilized to determine the model fit, including the goodness-of-fit index, the comparative fit index (CFI), the root mean squared error of approximation and the χ^2^/df. Table 4 shows the measures used to test these indices.

**Table 4:** Measures used to test the goodness of model fit [21]

Fit index	Good fit value	Acceptable value
$\chi^{2}$/df	≤3	≤4-5
GFI	≥0.90	0.89–0.85
CFI	≥0.97	≥0.95
RMSA	≤0.05	0.06–0.08

Relations between latent and measurement variables are depicted in the measurement model. Three constructs (latent variables) in the measurement model namely ergonomics, the efficiency of PPE, employee training are inter-related, as indicated by the two-headed arrows. Besides, 18 observed variables were enclosed in squares. The numbers on arrows depict the correlation coefficient between the latent variables and the standardized regression weights of the observed variables. The symbols e1– e15 are seen as errors in the measurement model.

**Fig. 2:**
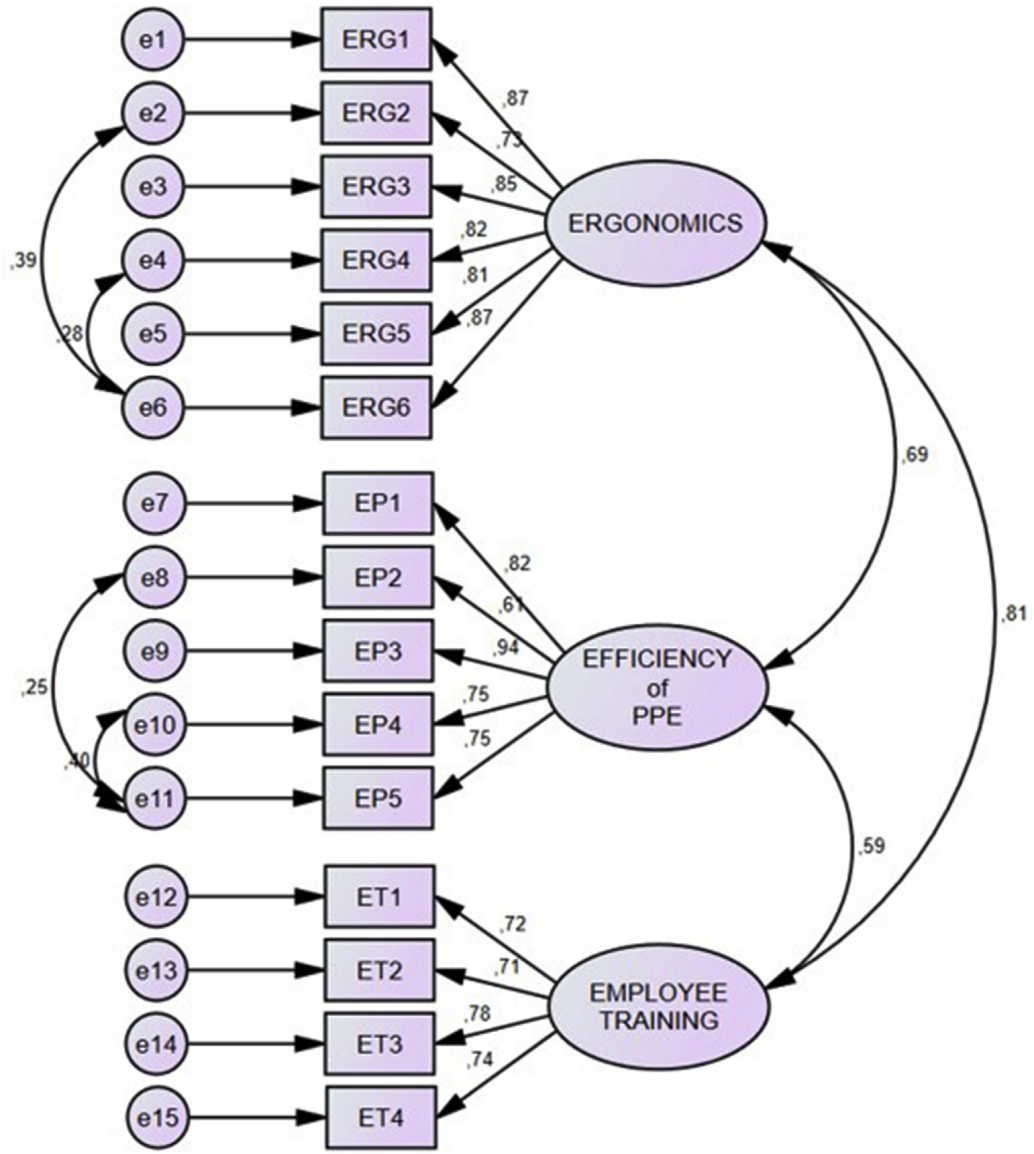
Modified measurement model.

Measurement model results were χ^2^/df = 1.688, goodness-of-fit index = 0.847, CFI = 0.942 and RMSA = 0.083. Results show that the initial model needed to be modified. Fig. 2 displays the outcome of the modified measurement model.

The modified measurement model results were χ^2^/df = 1.359, GFI = 0.883, CFI = 0.971 and RMSA = 0.060. Based on these results, all the goodness of fit values is in the acceptable range, which implied the validity of the modified measurement model.

### 2.5 Structural model

**Fig. 3:**
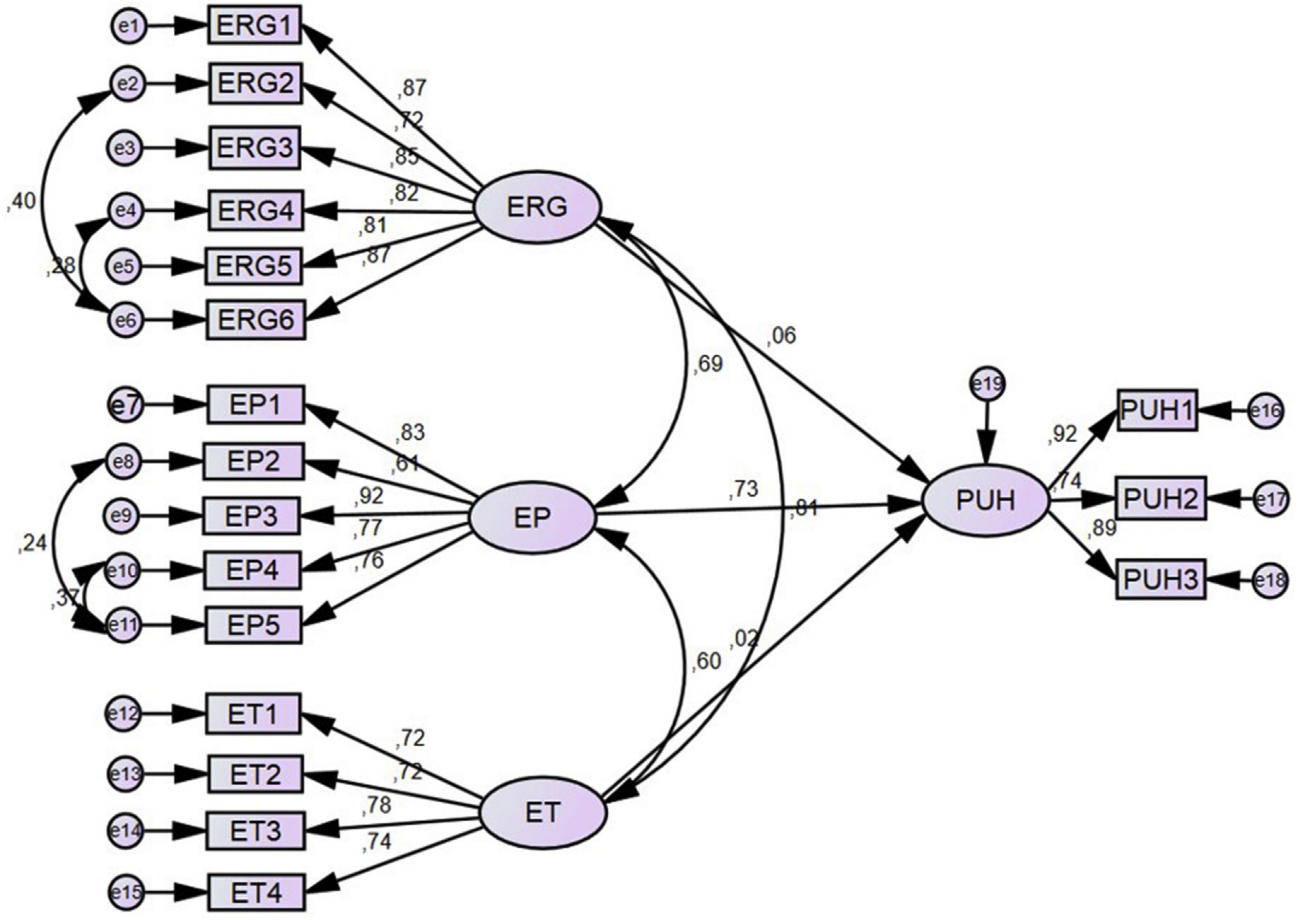
Structural model.

A common subject in the arrangement and determination of structural models is that an acceptable measurement model is desired before assessing a structural equation model [37]. According to these limitations, a model was constructed providing the goodness-of-fit in the study. Considering the impact of ergonomics (ERG), the efficiency of PPE (EP) and employee training (ET) constructs to the PPE usage habit (PUH), a structural model was suggested in Fig. 3. As a part of SEM, it is essential to improve the hypothesis demonstrating the correlation between the latent variables. Hypotheses of the study as follows:

H1 : Important positive relation presents between ergonomics and PPE usage habits.

H2 : Important positive relation presents between the efficiency of PPE and PPE usage habit.

H3 : Important positive relation presents between employee training and PPE usage habit.

The structural model results are χ^2^/df = 1.419, GFI = 0.856, CFI = 0.960, and RMSA = 0.065. The goodness of fit values is in a suitable range, which indicated a satisfactory fit between the structural model and the data. The results of the hypotheses were given in Table 5.

**Table 5:** Results of the hypotheses

Hypothesis	Path	*β*	*p*	Result
*H*_*1*_	ERG → PUH	0.064	0.713	Not supported
*H*_*2*_	EP → PUH	0.733	∗∗∗	Supported
*H*_*3*_	ET → PUH	0.022	0.889	Not supported

*β* = Standardized Regression Weights, *p* = Significant Probability. ∗∗∗ Significant probability value less than 0.001.

As the p values are higher than 0.05 in the relations of ERG and ET with the PPE use, it is determined that there is no statistically meaningful relationship. Thus, H_1_ and H_3_ hypotheses of the research were not supported. It has also resulted that the efficiency of PPE has a positive and statistically meaningful effect on PPE use (β = 0.733; p < 0.05). Hence, H_2_ hypothesis was supported based on this finding.

## 3 Results

The predicted positive influence of ERG on the PPE use (H_1_ hypothesis) was not supported because the ERG construct was not significantly related to the PPE use (p > 0.05). This indicates that ERG does not affect the PPE use of employees. Based on the research findings by Akbar-Khanzadeh et al. [14], despite all the huge stages in making PPE more attractive, better fitting or light-weight, approximately half of the employees searched did not admit their PPE as comfortable. Thus, safety managers need to provide more support including PPE manufacturers’ specifications and focus on the best likely products to achieve a particular safety task.

The predicted positive influence of the efficiency of PPE on the PPE use (H_2_ hypothesis) was supported because the efficiency of PPE showed a significant influence on PPE use (p < 0.05). This indicates that the efficiency of PPE affects the PPE use of employees. This result is relevant to a control system that has been conducted by safety managers. This control system showed a positive effect on PPE use. With the control program, the efficiency of the equipment is regularly provided according to the feedback from employees and accident analysis. On the other hand, specific drawbacks of using PPE such as disorder, exhaustion, and so on can be discovered in the very early stage. Jagt et al. [27] indicated the requirement of such a control program to improve the efficiency of PPE.

The predicted positive influence of ET on the PPE use (H_3_ hypothesis) was not supported because the ET construct was not significantly related to the PPE use (p > 0.05). This indicates that ET does not affect the PPE use of employees. Training should contain a component of theory along with practice in using the equipment and should be implemented compliant with the recommendations and directions provided by the PPE manufacturer. According to Newill et al. (1989), even those highly educated unsuccessful to use their PPE. Mayer and Korhonen [38] suggested focusing more on the proper selection of the devices and on the information and training of employees on the manner of wearing PPE. Lack of comfort is a major factor in the reported observation [6]. In a study by Murphy et al. [39], whether offered as the manufacturer’s printed directions, a short video training session specific to the product or as a one-on-one training session was evaluated using eight hearing groups and four hearing protection devices. After receiving individual instruction, poorly performing subjects were able to properly insert the earplugs and achieve sufficient attenuation. The focus of this study was to acquire more about the factors that influence employees' PPE usage patterns in a mining firm. The factors determined as part of the study's scope are specific to the operating conditions under which the study is conducted and will vary between enterprises. In the company, PPE usage training is held on a regular basis. The factor loading of the relevant item (ET2) reveals the outcome of regular training. Furthermore, the employees have a sufficient understanding of the work environment (Factor loading of ET1: 0.783). Thus, the factor load of the ET5 item was low as a result of this condition. When OHS knowledge is considered together with other characteristics, the study reveals that employees having appropriate information about risks and having enough PPE training are more efficient in forecasting usage behaviors.

## 4 Discussions

The prominence of the efficiency factor in the usage of PPE allowed a research topic to be combined: training alone may not be sufficient and, thus, the development of training programs should be conducted together with the efficiency of PPE. Both the design of vocational training programs and the prevention of occupational accidents in OHS programs benefit from the intersection of these two topics. This research is based not only on employee occupational health and safety training, but also on the impact of effectiveness on PPE usage, which is the ultimate goal of increasing PPE usage. This OSH learning model based on the activity-training theory is beneficial for assuring productivity in a work organization and generating long-term solutions that promote the development of job skills, including OHS components.

According to study results, education should be combined with other forms of intervention, such as engineering and ERG. To achieve a positive outcome, management's safety training, support, goal-setting, providing feedback to stimulate application of newly acquired knowledge, and incentives or prizes to reinforce performance are critical. Workers who have been trained to protect themselves from specific workplace dangers may still be damaged [40,41]. According to the findings of this study, education alone is ineffective in the usage of personal protective equipment. In addition to insufficient training provided to employees in the enterprises, employees' improper usage of personal protective equipment lowers the effectiveness of the PPE, even when it is chosen in accordance with norms and regulations. This condition has a negative impact on the utilization of personal protective equipment. A good training program can mitigate this harmful influence.

The factor load value is a coefficient that explains the link between the items and the factors. The load values of the factors in which the items occur are expected to be high. If a group of items is significantly linked with a factor, this indicates that the items as a whole measure the construct in issue. When an item's factor loading value is low, it means the item isn't highly related to the factor in question. The fact that an object has a low factor loading merely suggests that it is judged ineffective for measuring that construct. Keeping an unnecessary component in a model will have an impact on the model's fitness index in SEM [42,43]. Thus, the focus of this research is to find out which factors are important in the emergence of a PPE usage habit using a scale whose reliability and validity were verified via explanatory factor analysis. The model's starting point is pure theory, with the goal of giving a strong model fit index to correctly interpret the study results. The elements that affect construct validity were removed from the analysis in the literature and the model was tested [44].

## 5 Conclusions

Application of SEM was demonstrated to determine the efficient factors on PPE usage for employees particularly in the mining environment in the present study. Preventing occupational accidents can be achieved via the analysis of multiple factors simultaneously. Thus, it is essential to use a technique that regards multiple criteria to explain the problem such as SEM.

The results of the survey were analyzed using Exploratory and Confirmatory Factor Analysis. Finally, the structural model was constructed to examine the impact of ERG, efficiency of PPE and ET variables on the PPE use variable. The results of the hypotheses explain that the efficiency of PPE, with the factor loading of 0.733, is the most effective factor. ERG and ET factors are not efficient on the PPE use due to insignificant p values (p > 0.05).

Many studies have been carried out by various variables. However, none evaluated these variables simultaneously, especially in one model. The developed model in the present study enables some guidance to companies to better focus on ERG and ET to improve employees’ PPE usage. The PPE use can considerably be gained with consideration of combined ergonomic design and a detailed training program. Risks employees may encounter in case of not being used PPE should be emphasized in occupational health and safety training. Future studies need to develop the structural models in which employees actively participated in different sectors.

## Ethics approval

Ethical approval of the study was gained from the Ethics Committee of Eskisehir Osmangazi University with the number 2020-01.

## Conflict of Interest

On behalf of all authors, the corresponding author states that there is no conflict of interest.
